# Investigating the Relationship between Glaucoma Prevalence and Trabecular Meshwork Length

**DOI:** 10.3390/jcm10051096

**Published:** 2021-03-05

**Authors:** Wungrak Choi, Hyuna Cho, Eun Woo Kim, Hyoung Won Bae, Chan Yun Kim, Gong Je Seong

**Affiliations:** Institute of Vision Research, Department of Ophthalmology, College of Medicine, Yonsei University, Seoul 03722, Korea; wungrakchoi@hanmail.net (W.C.); hyunaa13@yuhs.ac (H.C.); OPHQ5@yuhs.ac (E.W.K.); baekwon@yuhs.ac (H.W.B.); kcyeye@yuhs.ac (C.Y.K.)

**Keywords:** anterior segment optical coherence tomography, ethnicity, glaucoma prevalence, trabecular meshwork

## Abstract

Glaucoma is one of the most common causes of blindness worldwide, but the risk factors of glaucoma are yet to be fully understood. We investigated the relationship between the prevalence of glaucoma and trabecular meshwork (TM) length by comparing the mean TM length of a South Korean population with that of another ethnic population. We included 250 eyes of 125 patients who underwent anterior segment optical coherence tomography at Yonsei University Gangnam Severance Hospital between January 2015 and December 2017. We measured the distance from the scleral spur to Schwalbe’s line in patients with open and closed angles and calculated the TM length using the open- and closed-angle ratios in the general population. The mean TM length of the patients in our study was 752 ± 116 μm. Considering the compensated data, the estimated true mean TM length in the Korean population was 793 ± 76 μm, which was similar to the mean TM length of a previously evaluated Hispanic population, but differed significantly from those of previously evaluated Asian (Chinese), Caucasian, and African-American populations (*p* < 0.05). Our results support the hypothesis that the development of glaucoma would be affected by TM length.

## 1. Introduction

Glaucoma is one of the most common causes of blindness worldwide [1]. However, evidence regarding the risk factors of glaucoma is still lacking, and it is not exactly known what causes its development [2]. The prevalence of glaucoma varies according to ethnicity [3,4,5,6]. In a previous report, African-American individuals were reported to have the highest prevalence of primary open-angle glaucoma (POAG; approximately 5.6%), followed by Hispanics (4.7%), Asians (2.4%), and Caucasians (1.7%) [7]. The present study attempted to explain the differences in the prevalence of POAG on the basis of differences in trabecular meshwork (TM) length.

Similar to POAG, primary angle-closure glaucoma (PACG) also exhibits ethnic differences in its prevalence, which is higher in Asian than in Western populations (African Americans, Hispanics, and Caucasians) [8]. Even in Asian populations, the prevalence of PACG differs by region and nationality, with higher incidence rates in Chinese populations than in people from other Asian countries [8].

Previous studies have measured TM size by anterior segment optical coherence tomography (AS-OCT) [7,9,10]. However, the results of these studies vary substantially by the study region and ethnicity of the participants [7]. Moreover, some studies have revealed differences in TM length between patients with open and closed angles [9,10].

Considering these factors, we hypothesized that the variation in TM length might contribute to the variation in the prevalence of glaucoma (both open- and closed-angle) by region and ethnicity. To investigate our hypothesis that differences in TM length may indicate differences in the incidence of glaucoma or risk of developing glaucoma among different ethnicities, it is important to know the correct mean TM length of a certain population.

The purpose of this study was to evaluate the relationship between the prevalence of glaucoma and TM length. We calculated the correct mean trabecular meshwork (TM) length of a South Korean population and compared this with the previously calculated mean TM length of other ethnic populations.

## 2. Materials and Methods

### 2.1. Patient Enrollment

This study was a retrospective, observational, single-center study. Among the patients who underwent AS-OCT at Yonsei University Gangnam Severance Hospital, Seoul, South Korea, between January 2015 and December 2017, 125 patients (250 eyes) were randomly enrolled in this study. Patients were selected using a random number generator. The medical records and results of complete ocular examination of these patients were systematically evaluated. Ocular examination findings included current ophthalmologic diagnosis, visual acuity, intraocular pressure (IOP), gonioscopy records, central corneal thickness (CCT), anterior chamber depth (ACD), axial length (AXL), and TM length. The exclusion criteria were as follows: history of intraocular surgery or penetrating trauma, corneal opacities that could hinder AS-OCT, and poor quality of AS-OCT images. Patients with a history of laser iridotomy were not excluded.

The Gangnam Severance Hospital institutional review board approved the study and provided a waiver of informed consent for the retrospective review of existing patient records (IRB number: 3-2018-0081). The methods used in this study adhered to the tenets of the Declaration of Helsinki and were HIPAA compliant.

### 2.2. Study Design

Closed angle was defined as an angle with less than 90° of the posterior trabecular meshwork visible by gonioscopic evaluation. In contrast, open angle was defined as an angle with more than 90° of the posterior trabecular meshwork visible. These definitions were based on those used in the previous NAMIL study [11]. We did not distinguish the closed-angle group as primary angle-closure suspect, primary angle-closure, PACG, or chronic angle-closure glaucoma. A trained ophthalmologist measured TM lengths on the nasal and temporal sides of the eye, which were manually chosen at the 3 and 9 o’clock positions, respectively. TM length was defined as the distance between Schwalbe’s line and the scleral spur. The baseline demographics and ocular examination results of the patients were masked from this specialist. The primary question in our study was whether the data from our patient population are representative of the entire South Korean population. Since this study was conducted at a tertiary hospital, the proportions of patients with open and closed angles might differ from those in the general South Korean population. Thus, we needed to exclude the possibility of selection bias during patient inclusion. To this end, we used the open- and closed-angle ratios of the general South Korean population, which had previously been reported in the NAMIL study [11]. The NAMIL study, a cross-sectional epidemiologic study of residents aged 40 years or above in Namil-myon, a rural area in central South Korea, is a representative population-based prevalence study in South Korea. According to the NAMIL study, the proportions of individuals with open and closed angles in Korea are 96.8% and 3.2%, respectively [11]. The TM lengths of patients with open and closed angles in the present study were weighted on the basis of these previously reported proportions. The calculated mean TM length of the present study population was also compared with those reported in previous foreign studies.

### 2.3. AS-OCT Measurements

All patients included in this study underwent AS-OCT at Yonsei University Gangnam Severance Hospital. Anterior segment images were obtained by swept-source OCT with a CASIA AS-OCT device (Tomey Corporation, Nagoya, Japan) using the anterior angle protocol. The participants were instructed to fixate their eye centrally for the scan. All measurements were performed by trained personnel. Thirty randomly selected eyes were reanalyzed to verify the reproducibility of TM length measurements.

Standard protocols for TM length measurement were used as described previously [12]. Briefly, TM length was measured in the nasal and temporal sides of the eye using AS-OCT by manually selecting 3 and 9 o’clock positions as the nasal and temporal positions, respectively. We used one image along the horizontal meridian, taken in the dark-light condition. We used normal-resolution scan mode for the results and the recently described band of extracanalicular limbal lamina (BELL) method to measure TM length [13]. TM length was defined as the distance between the scleral spur and Schwalbe’s line. The scleral spur was identified with the bump seen as an internal projection of the sclera into the anterior chamber or following the interface between the sclera and the ciliary muscle until it intersects a line projected along the inner cornea [14]. BELL was defined as a hypo-reflective band wrapping around the TM and Schlemm’s canal [13]. The location of Schwalbe’s line was defined as the corneal termination of a hypo-reflective band [13]. The location was confirmed using the tip of the U-shaped interface between the cornea and sclera [9,14]. Schwalbe’s line was assumed to be located at the inner apex of the U-shaped interface.

### 2.4. Statistical Analysis

Data were analyzed using the SPSS 22.0 (SPSS, Chicago, IL, USA), SAS (version 9.3; SAS Institute Inc., Cary, NC, USA), and R package (version 3.2.2; http://www.R-project.org, accessed on 20 January 2021) software and expressed as means ± standard deviations.

Differences between the groups were examined by an independent two-sample t-test. Linear regression analysis was used to calculate factors that affected the TM height. Variables included age, sex, IOP, spherical equivalent, AXL, CCT, and ACD. Variables that were significant and those that had been found to be significant in previous studies were analyzed with a multivariable model. The level of statistical significance was set at *p* < 0.05.

## 3. Results

We retrospectively reviewed the data of 250 eyes from 125 randomly selected patients who underwent AS-OCT at Yonsei University Gangnam Severance Hospital. For the final analysis, we included 245 eyes from 125 patients; 5 eyes were excluded because of poor image quality. Of the 245 eyes selected for inclusion, 194 were revealed to be open-angle and 51 were closed-angle. The reproducibility of TM length measurements was verified by the intraclass correlation coefficient (ICC). A comparison of the measurements from the 30 eyes randomly selected for reanalysis yielded an ICC of 0.91.

### 3.1. Baseline Characteristics

The baseline characteristics of the study population are summarized in Table 1. The mean age of the patients was 52.9 years, and the mean baseline IOP was 13.9 mmHg. The mean ACD and CCT were 2.78 ± 0.57 mm and 540.2 μm, respectively (Table 1). The baseline characteristics for each open and closed groups are defined in Supplemental Table S1.

### 3.2. Mean Trabecular Meshwork Length in the South Korean Sample Population

The mean TM length of the patients in our study was 752 ± 116 μm. The mean TM lengths in the open- and closed-angle groups were 799 ± 62 μm and 573 ± 96 μm, respectively (Table 2). To calculate the true TM length of the general South Korean population, the ratios of the open- and closed-angle groups were adjusted to 96.8% and 3.2%, respectively, as reported in the NAMIL study [11]. The compensation equation was as follows: 799 μm × 96.8/100 + 573 × 3.2/100. After compensation, the estimated value of the true mean TM length of the general South Korean population was 792 ± 76 μm.

### 3.3. Factors Affecting Trabecular Meshwork Length

In the linear regression analysis, patient age, baseline IOP, ACD, spherical equivalent, AXL, and sex were found to be the factors that significantly affected TM length. Multivariate analysis was performed to calibrate these demographic characteristics, and the results revealed spherical equivalent and ACD to be the factors affecting the mean TM length (Table 3). As only 23 eyes had AXL data, due to the lack of enough data, AXL was not used for multivariate analysis.

### 3.4. Comparison of Trabecular Meshwork Length between South Koreans and Other Ethnic Populations

Next, we compared our data on TM length with the data previously reported in a study that had investigated ethnic differences in TM length in America [7]. Since this previous study solely evaluated the nasal TM length, we only compared the data on nasal TM length between the two studies.

A comparison of the non-compensated raw data revealed that TM lengths of the South Korean population were significantly less than those of Caucasian, Asian, and Hispanic populations and similar to those of African-Americans. Even after comparing the compensated data of the general South Korean population, TM lengths of the South Koreans were significantly less than those of Caucasian and Asian populations, and were similar to those of Hispanic and African-American populations.

However, because of the differences in open- and closed-angle ratios reported in the previous study in America, we finally compensated the open- and closed-angle ratios of the South Korean patients with those of each ethnic group in order to perform a more precise comparison. The results showed that TM lengths of the South Korean patients were less than those of Caucasian and Asian populations, similar to those of Hispanic populations, and greater than those of African-American populations (Table 4).

### 3.5. Comparison of Trabecular Meshwork Length between South Koreans and Other Asian Populations

We compared the TM length data calculated in the present study with those previously reported in a study conducted in Singapore [9]. Overall, the mean TM lengths of South Korean patients were greater than those of Singaporean-Chinese patients on both the nasal and temporal sides. Specifically, South Koreans in the open-angle group had greater TM lengths compared to those of their Singaporean-Chinese counterparts. However, a contrasting trend was noted in the closed-angle group, whereby the Singaporean-Chinese patients had greater TM lengths than their South Korean counterparts. There were significant differences between the two populations in terms of both open- and closed-angle TM lengths (Table 5).

## 4. Discussion

A previously published study reported that TM length could affect the prevalence of glaucoma [7]. The authors noted that populations with a lesser TM length showed a higher incidence of POAG [7]. This was a valuable study that put forth the idea that TM length might contribute to the development of glaucoma.

In the present study, our goal was to evaluate the previously suggested theory that there might be an association between the prevalence of glaucoma and TM length by comparing TM lengths of a South Korean population with those of other ethnic populations. Based on previous studies that have reported differences in TM length between open- and closed-angle patients, we compensated the TM length data of the South Korean patients with the corresponding open- and closed-angle ratios of each ethnic group in the previously discussed American study for a more accurate comparison [10,12].

We observed that mean TM lengths of South Koreans were less than those of Caucasian and Asian populations, similar to those of Hispanics, and greater than those of African-Americans. Meanwhile, the prevalence of POAG among South Koreans is 2.6–4.7%, which is similar to that among Hispanics (4.7%), lower than that among African-Americans (5.6%), and greater than that among Caucasian (1.7%) and Asian (2.4%) populations [7,15,16,17]. Taken together, our results support the hypothesis that the prevalence of glaucoma may be affected by TM length (Table 4).

On the other hand, the data on Asian populations in the previous study varied substantially from our results. There may be several reasons for this variation. First, the present and previous studies used different OCT techniques. While we used swept-source AS-OCT in the present study, the previous America study used spectral-domain OCT. Second, since neither study was conducted on a large scale, there is a possibility of selection bias. Third, we measured TM length on the nasal and temporal sides of the eye; the previous America study only measured the nasal TM length. Fourth, the previous study did not calculate the actual population distribution and characteristics of open- and closed-angles. The distribution of closed- and open-angles can differ substantially even within the same Asian population [8,18]. Finally, even though all Asian individuals in the previous study were classified under the same group, there may have been differences in results depending on their nationalities and regions.

To increase the accuracy of our analysis, we compared the present TM length data with those of a previous Asian study involving Singaporean-Chinese individuals [19]. We categorized the patients by angle configuration (open or closed) and compared TM lengths on the nasal and temporal sides (Table 5). The results revealed significant differences between the South Korean and Singaporean-Chinese patients not only in the overall mean TM length but also in both open- and closed-angle TM lengths. This result implies that even among individuals of the same Asian ethnicity, subpopulations should be distinguished more specifically by region or nationality when comparing anterior segment structures.

Although our results lend credence to the hypothesis that TM length affects the prevalence of glaucoma, it cannot be said that TM length is the only factor that can lead to the development of glaucoma. However, since glaucoma is assumed to be a multifactorial disease, it is reasonable to assume that TM, which plays a key role in regulating IOP, might have some influence on the onset of glaucoma.

It is widely known that anterior chamber depth is affected by race and ethnicity, and since AXL, ACD, and spherical equivalents are all closely related to each other, it can be assumed that TM length will be influenced by these factors [12,20,21,22]. It is also widely discussed about the prevalence of glaucoma (especially angle closure glaucoma) associated with different ethnicities with different angular parameters, including AXL, ACD, and spherical equivalents [7,8,18,21,22]. Comprehensively, the different of TM length which is affected by AXL, ACD, and spherical equivalents may be one of the causes of racial differences in glaucoma prevalence. However, this aspect of view needs to be studied in future for confirmation.

There may be several reasons why TM length can increase the risk of development of glaucoma. The TM is the core structure that controls IOP by regulating the outflow of aqueous humor [23]. TM function is essential for maintaining proper aqueous outflow [23]. It can be assumed that a short TM length indirectly indicates a smaller TM. Compared to a larger TM, a smaller one may have to overwork to maintain proper outflow and, therefore, more prone to stress. Moreover, a smaller TM may be weaker against the debris that can accumulate around the TM and Schlemm’s canal. These aspects should be investigated in greater depth in future studies.

The novelty of our study is that we attempted to calculate the true mean TM length of the whole South Korean population on the basis of actual open- and closed-angle population ratio results. In the NAMIL study, which involved residents in a rural area in central South Korea, the prevalence rates of open- and closed-angles were 96.8% and 3.2%, respectively [11]. The average TM length of the 245 randomly selected eyes in the present study was 752.109 μm. However, when the data were weighted in accordance with the true distribution of angle configurations in the actual population, the compensated mean TM length was 791.981 μm, indicating approximately a 5.3% difference between the two results (Table 2). We can gather from our results that when estimating mean TM lengths in a specific condition or region, the data should be calculated according to the actual open- and closed-angle ratios in the population.

The limitations of our study include its retrospective design and indirect methodology for comparing TM lengths of South Koreans with those of other ethnicities and populations. When the results are compared with other ethnic groups, age, sex, and other important factors should be considered. However, due to the lack of previous study data, such a comparison was not possible. Furthermore, the data of comparison between normal subjects and glaucoma patients are not included in this study. To validate our results, it would be helpful to compare the TM lengths of not just two but numerous other countries and ethnicities. Further prospective cross-sectional studies should be conducted to determine the true difference in mean TM lengths among Asian populations.

In the present study, we also analyzed the baseline characteristics of the study population to identify the factors that affect TM length. The results of the multivariate analysis revealed spherical equivalent and ACD to be the factors affecting the mean TM length (Table 3). Unfortunately, our study was retrospective in nature, and we were unable to perform multivariate analysis with AXL due to the lack of enough AXL data. A large-scale prospective study should be conducted to investigate this aspect in the future.

In conclusion, the results of the comparison of our data with the mean TM lengths of other ethnic populations supported the opinion that the development of glaucoma can be affected by TM length.

## Figures and Tables

**Table 1:** Baseline clinical characteristics.

Characteristic	Mean ± SD or *n* (%)
Age (years)	52.96 ± 16.07
Sex (no. of eyes)	
Male	115 (46.94%)
Female	130 (53.06%)
IOP (mmHg)	13.93 ± 4.01
Spherical equivalent (D)	−1.72 ± 4.02
Anterior chamber depth (mm)	2.78 ± 0.57
CCT (µm)	540.23 ± 33.18

IOP = intraocular pressure; SD = standard deviation; CCT = central corneal thickness.

**Table 2:** Mean trabecular meshwork height in South Koreans.

Variable	Mean ± SD
Non-Compensated Data	
Total (N = 245)	
Nasal and temporal mean	752 ± 116
Nasal	762 ± 118
Temporal	744 ± 127
Open-angle group (*n* = 194)	
Nasal and temporal mean	799 ± 62
Nasal	804 ± 77
Temporal	794 ± 77
Closed-angle group (*n* = 51)	
Nasal and temporal mean	573 ± 96
Nasal	594 ± 106
Temporal	560 ± 104
Compensated Data (Weighted Average for Open-Angle: 96.8%; Closed-Angle: 3.2%)	
Nasal and temporal mean	792 ± 76
Nasal	798 ± 75
Temporal	786 ± 76

SD = standard deviation. Weighted average: $\bar{x}=\frac{\sum w_{i}x_{i}}{\sum w_{i}}$, $x_{i}$: *i* observation value, $w_{i}$: weight of the *i* observation value. Weight: ratio of the total population/ratio in data. Weight in the open-angle group: 0.968/0.7918 = 1.2225; weight in the closed-angle group: 0.032/0.2082 = 0.1537.

**Table 3:** Linear regression analysis of the factors affecting trabecular meshwork (TM) height.

Variable	Mean TM Height			
	Univariable		Multivariable	
	*B (SE)*	*p* Value	*B (SE)*	*p* Value
CCT	−0.228 (0.242)	0.3468	0.112 (0.200)	0.5759
Age	−2.396 (0.440)	<0.001	0.222 (0.468)	0.6361
IOP	−3.725 (1.875)	0.0481	−0.975 (1.555)	0.5314
Spherical equivalent	−12.318 (1.896)	<0.001	−6.104 (1.647)	<0.001
AXL	39.837 (7.176)	<0.001		
ACD	142.296 (9.615)	<0.001	127.434 (13.322)	<0.001
Sex				
M	ref (0)		ref (0)	
F	−34.537 (14.690)	0.0195	5.088 (13.501)	0.7068

TM = trabecular meshwork; CCT = central corneal thickness; IOP = intraocular pressure; SE = standard error; AXL = axial length; ACD = anterior chamber depth. Univariable and multivariable linear regression analyses were performed. Multivariable data were obtained as a result of adjusting all the variables accordingly. Due to the lack of enough data, AXL was not used for multivariate analysis.

**Table 4:** Comparison of trabecular meshwork height among various ethnicities.

Variable	Korean Study		American Study [7]											
	Korean (1)		White (2)		Asian (3)		Hispanic (4)		African American (5)		*p*-Value			
	*n*	TM (µm) Mean ± SD	*n*	TM (µm) Mean ± SD	*n*	TM (µm) Mean ± SD	*n*	TM (µm) Mean ± SD	*n*	TM (µm) Mean ± SD	(1) vs. (2)	(1) vs. (3)	(1) vs. (4)	(1) vs. (5)
Non-compensated data	245	752 ± 116	157	851 ± 131	203	843 ± 126	55	822 ± 147	45	771 ± 118	<0.001	<0.001	<0.001	0.317
Compensated data	245	791 ± 76	157	851 ± 131	203	843 ± 126	55	822 ± 147	45	771 ± 118	<0.001	<0.001	0.031	0.123
Compensation of open and closed angle ratios		799 ± 84	118/39	851 ± 131							<0.001			
		801 ± 85			130/73	843 ± 126						<0.001		
		802 ± 116					30/25	822 ± 147					0.183	
		791 ± 76							32/13	771 ± 118				0.049

Our data on the TM heights of South Koreans were compared with previously reported American data which studied the TM height based on ethnicity (nasal TM height). Non-compensated data is the mean nasal TM height of 242 eyes. Compensated data is the mean weighted average TM height (Open angle: 96.8%, closed: 3.2%). Due to the different ratios of open- and closed-angle in the American study, open- and closed-angle ratios of South Korean patients were compensated. *p*-value was calculated using an independent two sample t-test. TM: Trabecular meshwork.

**Table 5:** Comparison of TM heights of South Koreans with previously reported data in the Singaporean study.

Variable	South Korean Study		Singapore Study [9]		*p* Value
	*n*	TM (µm) Mean ± SD	*n*	TM (µm) Mean ± SD	
Total (open- and closed-angle)					
Nasal	242	762 ± 118	141	712 ± 137	<0.001
Temporal	242	744 ± 127	141	724 ± 115	0.119
Open-angle group					
Nasal	194	804 ± 77	102	717 ± 142	<0.001
Temporal	191	794 ± 77	102	734 ± 116	<0.001
Closed-angle group					
Nasal	48	594 ± 106	39	698 ± 122	<0.001
Temporal	51	560 ± 104	39	696 ± 108	<0.001

TM = trabecular meshwork; SD = standard deviation.

## Data Availability

The datasets used and/or analyzed during the current study are available from the corresponding author on reasonable request.

## Supplementary Materials

The following are available online at https://www.mdpi.com/2077-0383/10/5/1096/s1, Table S1: Baseline clinical characteristics of open and closed angle groups.
