# Supplementary material for: Investigating the Relationship between Glaucoma Prevalence and Trabecular Meshwork Length
Source: J Clin Med. 2021 Mar 5;10(5):1096. doi: 10.3390/jcm10051096 (PMC7961563; doi:10.3390/jcm10051096)
Supplement: Supplementary file 1 [file jcm-10-01096-s001.pdf]

Supplementary Table S1. Baseline clinical characteristics of open and closed angle groups

Characteristic	Mean ± SD or *n* (%)	
	**Open closed**	
Age (years)	50.00 ± 16.16	64.10 ± 9.64
Sex (no. of eyes)		
Male	99 (51.03%)	16 (31.37%)
Female	95 (48.97%)	35 (68.63%)
IOP (mmHg)	13.50 ± 3.21	15.51 ± 5.90
Spherical equivalent (D)	−2.71 ± 3.51	1.54 ± 3.91
Anterior chamber depth (mm)	3.02 ± 0.33	1.89 ± 0.34
CCT (μm)	539.15 ± 2.55	543.92 ± 34.03

IOP = intraocular pressure; SD = standard deviation; CCT = central corneal thickness
